# A Genome-Wide Association Study Identifies rs2000999 as a Strong Genetic Determinant of Circulating Haptoglobin Levels

**DOI:** 10.1371/journal.pone.0032327

**Published:** 2012-03-05

**Authors:** Philippe Froguel, Ndeye Coumba Ndiaye, Amélie Bonnefond, Nabila Bouatia-Naji, Aurélie Dechaume, Gérard Siest, Bernard Herbeth, Mario Falchi, Leonardo Bottolo, Rosa-Maria Guéant-Rodriguez, Cécile Lecoeur, Michel R. Langlois, Yann Labrune, Aimo Ruokonen, Said El Shamieh, Maria G. Stathopoulou, Anita Morandi, Claudio Maffeis, David Meyre, Joris R. Delanghe, Peter Jacobson, Lars Sjöström, Lena M. S. Carlsson, Andrew Walley, Paul Elliott, Marjo-Riita Jarvelin, George V. Dedoussis, Sophie Visvikis-Siest

**Affiliations:** 1 Centre National de la Recherche Scientifique (CNRS) 8199 - Institute of Biology, Pasteur Institute, Lille 2 University, Lille, France; 2 Genomic Medicine, Imperial College London, Hammersmith Hospital, London, England; 3 EA4373–‘Cardio-vascular Genetics’ Research Unit, Université de Lorraine, Nancy, France; 4 Institut National de la Santé et de la Recherche Médicale (INSERM) U954, Faculté de Médecine, Nancy-Université, Nancy, France; 5 Department of Clinical Chemistry, Ghent University Hospital, Ghent, Belgium; 6 Department of Cardiovascular Diseases, Faculty of Medicine and Health Sciences, Ghent University, Ghent, Belgium; 7 Institute of Clinical Medicine/Biochemistry, University of Oulu, Oulu, Finland; 8 Regional Centre for Juvenile Diabetes, Obesity and Clinical Nutrition, Verona, Italy; 9 Department of Molecular and Clinical Medicine, The Sahlgrenska Academy, University of Gothenburg, Gothenburg, Sweden; 10 Department of Epidemiology and Biostatistics, MRC Health Protection Agency (HPA) Centre for Environment and Health, School of Public Health, Imperial College London, London, England; 11 Institute of Health Sciences, Biocenter Oulu, University of Oulu, Oulu, Finland; 12 Department of Children, Young People and Families, National Institute for Health and Welfare, Oulu, Finland; 13 Department of Nutrition–Dietetics, Harokopio University, Athens, Greece; 14 Department of Internal Medicine and Geriatrics, ‘Centre Hospitalier Universitaire de Nancy’, Nancy, France; Johns Hopkins University, United States of America

## Abstract

Haptoglobin is an acute phase inflammatory marker. Its main function is to bind hemoglobin released from erythrocytes to aid its elimination, and thereby haptoglobin prevents the generation of reactive oxygen species in the blood. Haptoglobin levels have been repeatedly associated with a variety of inflammation-linked infectious and non-infectious diseases, including malaria, tuberculosis, human immunodeficiency virus, hepatitis C, diabetes, carotid atherosclerosis, and acute myocardial infarction. However, a comprehensive genetic assessment of the inter-individual variability of circulating haptoglobin levels has not been conducted so far.

We used a genome-wide association study initially conducted in 631 French children followed by a replication in three additional European sample sets and we identified a common single nucleotide polymorphism (SNP), rs2000999 located in the *Haptoglobin* gene (*HP*) as a strong genetic predictor of circulating Haptoglobin levels (*P_overall_* = 8.1×10^−59^), explaining 45.4% of its genetic variability (11.8% of Hp global variance). The functional relevance of rs2000999 was further demonstrated by its specific association with *HP* mRNA levels (β = 0.23±0.08, *P* = 0.007). Finally, SNP rs2000999 was associated with decreased total and low-density lipoprotein cholesterol in 8,789 European children (*P_total cholesterol_* = 0.002 and *P_LDL_* = 0.0008).

Given the central position of haptoglobin in many inflammation-related metabolic pathways, the relevance of rs2000999 genotyping when evaluating haptoglobin concentration should be further investigated in order to improve its diagnostic/therapeutic and/or prevention impact.

## Introduction

Human haptoglobin (Hp) is an acute phase inflammatory glycoprotein essentially synthesized by the liver and up-regulated by cytokines [1]. Hp is polymorphic with two co-dominant alleles, *Hp1* and *Hp2* encoded by the *Haptoglobin* (*HP*) gene located in chromosome 16 and resulting in three common isoforms: Hp1-1, Hp2-2 and Hp2-1 (called *HP* ‘common polymorphism’) [2]. In normal physiological conditions, Hp protein concentration in blood ranges between 0.3 and 2.0 g/L in adults [3] but significant fall in its level during the first decade of life [4]. The main property of Hp is to scavenge circulating hemoglobin (Hb) released by hemolysis or normal red blood cells turnover [5]. The resulting circulating Hp-Hb complexes are eliminated by Kupffer's cell(s) in the liver, preventing the generation of reactive oxygen species [2], [6]. Therefore, Hp plays an important role in preventing renal damage and iron loss that can occur following an intravascular hemolysis. Hp is also able to bind apolipoprotein (Apo) A-I [7] to protect the Apo A-I effector domain of lecithin-cholesterol acyltransferase against oxidative stress, and Hp consequently modulates the high-density lipoprotein (HDL) function [8]. Furthermore, Hp can bind Apo E and the resulting complexes influence cholesterol esterification [9]. These functional characteristics confer to Hp a major role in the reverse transport of cholesterol between peripheral cells and the liver for degradation.

Hp levels and *HP* rs72294371 ‘common polymorphism’ (Data S1) have been consistently associated with inflammatory-linked infectious [10], [11] and non-communicable diseases [11], [12]. Malaria caused by *Plasmodium falciparum*, which is associated with extensive intravascular hemolysis, decreases Hp to undetectable levels as the Hb-scavenging system is saturated [13].^13^ In malaria-endemic areas, hypohaptoglobinemia has been proposed as an indirect biochemical indicator of malaria [14]. *HP* ‘common polymorphism’ should be considered at diagnosis of tuberculosis. Eisaev and colleagues [15] described an increased recurrence of pulmonary tuberculosis with worse prognosis in Hp2-2 Caucasians. Furthermore, this *HP* ‘common polymorphism’ contributes to mortality and viral load in Human immunodeficiency virus (HIV) infection [16]. Hp2-2 HIV carriers have a more pronounced viral replication rate and a worse prognosis compared to Hp1-1 or Hp2-1 HIV carriers [16], [17]. Hepatitis C infection has also been associated with low serum Hp concentrations [18] and an overrepresentation of the Hp1-1 phenotype has been associated with high risk for chronic hepatitis C [19], [20]. The *HP* ‘common polymorphism’ has also an effect on various other infectious diseases [21], [22], [23], [24].

In Type 2 diabetes patients, the Hp2-2 phenotype has been suggested to confer greater risk of cardiovascular events [12], [25], [26] and of carotid atherosclerosis [27]. Moreover, high Hp level is a risk factor for acute myocardial infarction, stroke and heart failure [28], [29]. Therefore, the routine measurement of Hp level has been suggested to be incorporated in daily medical practice to evaluate cardiovascular risk [29].

Despite these findings, the basis of Hp level inter-individual variability is still unknown. To identify genetic variants modulating physiological levels of Hp, we analyzed genome-wide association study (GWAS) data generated in European children for whom no age-related disease may influence Hp concentrations. We also assessed the association between the identified variants and cardiovascular risk factors (total, HDL and low density lipoprotein-cholesterol, Apolipoproteins A1 and B).

## Results

Table 1 shows the phenotypic characteristics of the studied populations. Hp levels in children were low, in accordance with its reference distribution [4].

**Table 1:** Phenotypic characteristics of the studied populations.

Population	Subsample	Sample size (% female)	Age (years) [95% CI]	BMI (kg/m^2^) [95% CI]	Haptoglobin (g/L) [95% CI]	LDL-cholesterol (mmol/L) [95% CI]	Total cholesterol (mmol/L) [95% CI]
SFS	Discovery cohort: Phase 1 vs Hp	631 (50.9%)	11.93 [11.76–12.11]	17.66 [17.49–17.84]	0.65 [0.62–0.68]	2.99 [2.94–3.05]	4.79 [4.74–4.85]
	Families: Phase 2 vs Hp and lipids	2,957 (49.2%)	29.84 [29.38–30.30]	22.66 [22.52–22.81]	0.95 [0.94–0.97]	3.43 [3.40–3.46]	5.30 [5.26–5.34]
Obese children	Replication cohort: Phase 2 vs Hp and lipids	1,015 (52.3%)	11.07 [10.86–11.27]	28.24 [27.84–28.64]	1.18 [1.15–1.22]	2.74 [2.70–2.79]	4.47 [4.42–4.52]
GENDAI	Replication cohort: Phase 2 vs Hp and lipids	419 (53.1%)	11.16 [11.10–11.23]	19.76 [19.46–20.07]	0.81 [0.76–0.85]	3.12 [3.06–3.17]	4.79 [4.73–4.86]
NFBC1986	Replication cohort: Phase 2 vs lipids	5,310 (50.8%)	16	21.27 [21.17–21.37]	NA	2.54 [2.52–2.56]	4.27 [4.24–4.29]
VERONA cohort	Replication cohort: Phase 2 vs lipids	401 (43.4%)	10.90 [10.75–11.04]	18.01 [17.78–18.23]	NA	2.41 [2.40–2.47]	4.21 [4.15–4.28]

CI: confidence interval; BMI: body mass index; LDL: low-density lipoprotein.

Patterns of family correlations for serum Hp concentrations were assessed following both unadjusted values (Table 2). Model 1, which was not adjusted, did not show any family correlation. Model 2, which took into account age and body mass index (BMI) as covariates showed significant correlations for all the various pairs of relatives. Model 3, which hypothesized no effect of gender on family correlations, showed significant father-mother, father-son and son-son correlations (Table 2).

**Table 2:** Estimates of familial correlations ± standard error for serum haptoglobin concentration (656 familles/2680 individuals).

	Model 1	Model 2	Model 3	Model 4
Adjustment for	None	Age & BMI	Age & BMI	Age, BMI & rs2000999 allelic frequency
Father - Mother (FM)	0.126±0.038	0.111±0.038**	0.111±0.038**	0.156±0.038***
Father - Son (FS)	0.277±0.037	0.264±0.037***	0.230±0.021***	0.206±0.022***
Father - Daughter (FD)	0.277±0.036	0.265±0.037***	[0.230]	[0.206]
Mother - Son (MS)	0.227±0.036	0.204±0.037***	[0.230]	[0.206]
Mother - Daughter (MD)	0.184±0.039	0.188±0.040***	[0.230]	[0.206]
Son - Son (SS)	0.213±0.060	0.186±0.064**	0.274±0.036***	0.239±0.036***
Son - Daughter (SD)	0.273±0.047	0.271±0.047***	[0.274]	[0.239]
Daughter - Daughter (DD)	0.355±0.067	0.386±0.064***	[0.274]	[0.239]
Log_e_ L^2^ (Logarithm of likelihood function)	1458.83	1338.20	1342.20	1215.13
Alternate model		Model 1	Model 2	Model 3
χ^2^ (df)		241.26(8)	8.00(5)	254.14(4)
P		***	NS	***

* *P*≤0.05, ** *P*≤0.01, *** *P*≤0.001: compared to zero.

Model 1 estimated all eight correlations without adjustment.

Model 2 estimated all eight correlations with adjustment for age and BMI,

Model 3 estimated three correlations with adjustment for age and BMI with no gender effect on parent or offspring correlations: FS = MS = MD = FD and SS = SD = DD.

Model 4 estimated three correlations with adjustment for age, BMI and rs2000999 allelic frequency with no gender effect on parent or offspring correlations: FS = MS = MD = FD and SS = SD = DD.

Values in brackets were constrained to be equal to a preceding value according to the hypotheses of the model.

BMI: body mass index.

We then assessed the components of variance attributable to additive genetic effects, shared household effects and residual environmental factors (including assay variability) in 656 nuclear bi-parental families (2,680 individuals) from the STANISLAS Family Study (SFS) cohort (Table 3). Model 2, which included the three components after adjustment for age and BMI gave a better description of the variance decomposition than model 1 which was not adjusted. Hp genetic variance represented 26% (*P*<0.001) of the total variance. Shared (*i.e.* within families) and random environmental variances were 11.6 and 62.4% respectively (Table 3).

**Table 3:** Variance components of serum haptoglobin concentrations (656 families/2680 individuals, rs2000999 & rs4788597 as allelic frequency).

	Model 1	Model 2	Model 3
Adjustment for	None	Age & BMI	Age, BMI & rs2000999 allelic frequency
Polygenic variance: σ^2^_G_	0.0478±0.0120***, 24.9%	0.0460±0.0109***, 26.0%	0.0226±0.0101*, 14.2%
Household variance: σ^2^_H_	0.0250±0.0065***, 13.0%	0.0206±0.0059***, 11.6%	0.0233±0.0053***, 14.7%
Residual variance: σ^2^_E_	0.1194±0.0078***, 62.1%	0.1103±0.0072***, 62.4%	0.1130±0.0069***, 71.1%
Log_e_ L^2^ (Logarithm of likelihood function)	−968.90	−1074.58	−1200.04
Alternate model	-	Model 1	Model 2
χ^2^ (df)	-	211.36 (8)	250.92 (4)
P	-	***	***

* *P*≤0.05,

** *P*≤0.01,

*** *P*≤0.001.

BMI: body mass index.

**Figure 1:**
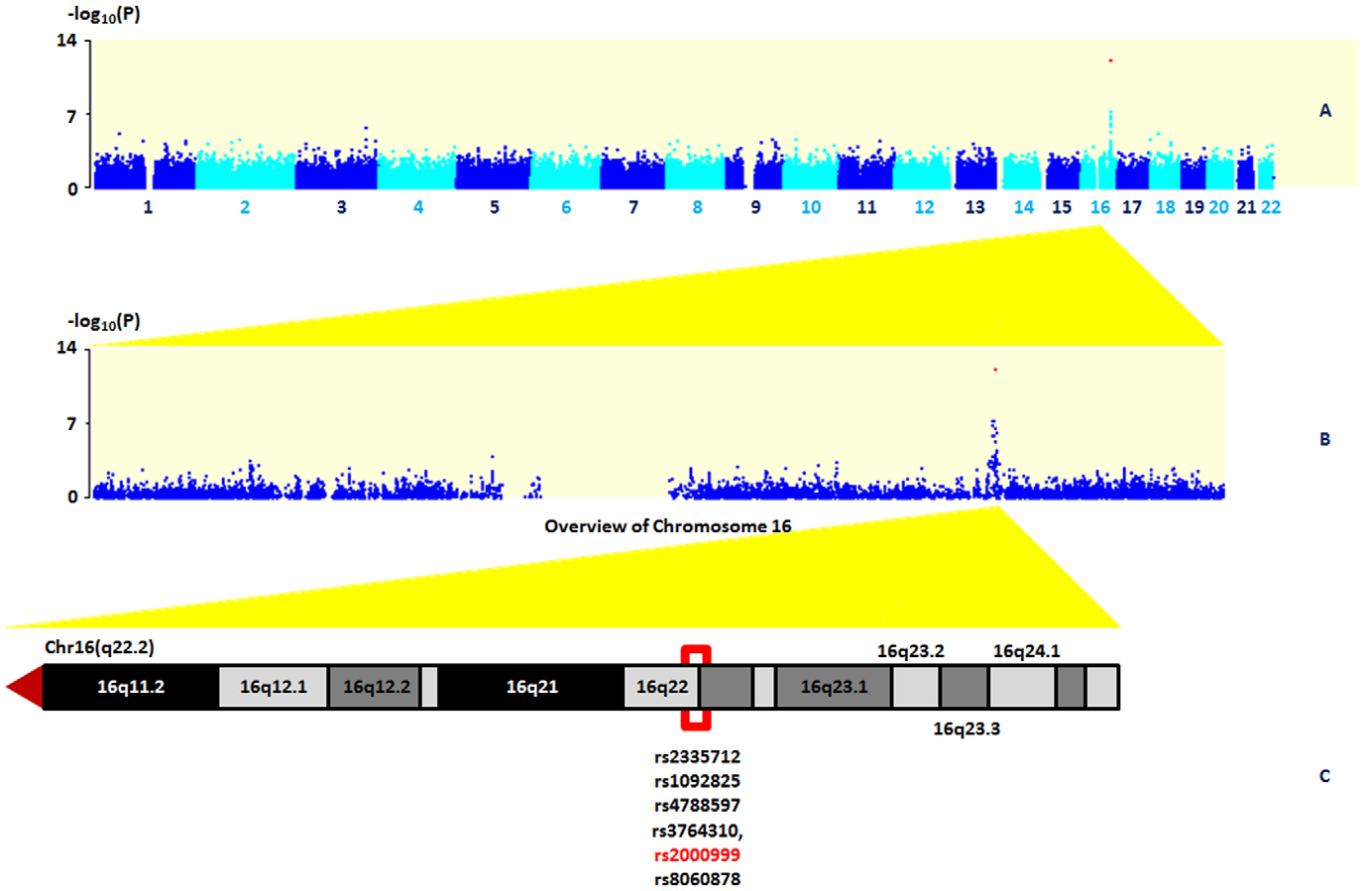
Manhattan plot of the GWAS of the discovery cohort comprising 631 children. **A**, A Manhattan plot showing the −log_10_(*P* values) of SNPs from the association analysis of the 631 SFS children from stage 1. **B**, An overview of the −log_10_(*P* values) of Chromosome 16. **C**, The genomic region of the 618 LD block displayed in UCSC Genome Browser.

Our GWAS based on 631 unrelated children of the SFS cohort showed strongest association signal for Hp levels in a 218-kb linkage disequilibrium (LD) block on chromosome 16 that includes the *HP* gene (Figure 1). Using the square-root transformed Hp measurement adjusted for gender, age and z-BMI under the additive model, we identified in this region two significant association signals 90-kb apart: rs2000999 (with A as allele effect: β = −0.123, standard error [SE] = 0.017, *P* = 6.32×10^−13^; Table 4) and rs10492825 (with C as effect allele: β = −0.0876, SE = 0.016, *P* = 5.50×10^−08^; Table 4). Both SNPs rs2000999 and rs10492825 display moderate LD (r^2^ = 0.48, HapMap CEU release #27). In order to assess the redundancy between these two signals, we ran conditional regression analyses for both SNPs adjusted for each other and found that rs2000999 alone drove the association observed at the *HP* locus (rs2000999: *P_rs10492825 adjusted_* = 1.95×10^−7^, rs10492825: *P_rs2000999 adjusted_* = 0.91, Data S2, Table S1).

**Table 4:** Discovery and replication of rs2000999 association data for Hp levels.

					Haptoglobin (g/L)					
Population	Stage	N	MAF	GG	GA	AA	β	Standard error	raw P	P adjusted with rs10492825
SFS - unrelated children	Discovery	631	0.21	0.736±0.406	0.532±0.321	0.361±0.180	−0.123	0.017	6.32×10^−13^	1.95×10^−07^
SFS - families	Replication	2,957	0.20	1.056±0.467	0.819±0.445	0.549±0.330	−0.138	0.007	1.17×10^−75^	
Obese children	Replication	1,015	0.22	1.261±0.492	1.064±0.455	1.002±0.534	−0.088	0.013	1.20×10−^11^	
GENDAI	Replication	419	0.25	0.811±0.450	0.813±0.558	0.731±0.319	−0.021	0.021	0.306	
Replication sample	Replication	4,391	0.22						3.49×10^−41^	
Overall meta-analysis	Replication	5,022							8.09×10^−59^	

N: sample size; MAF: Minor Allele Frequency; β:beta coefficient for the effect allele A.

We confirmed the association between SNP rs2000999 and circulating Hp levels in three additional independent European cohorts: the GENDAI study of Greek children, a subset of obese children from the East of France (*N_total_* = 1,434), and a familial subset (*N_total_* = 2,957) of the SFS cohort (*P_replication_* = 3.49×10^−41^, *P_overall_* = 8.09×10^−59^; Table 4).

Accounting for the rs2000999 allelic frequency, the pattern of familial correlation (Table 2, model 4) decreased from 0.230 to 0.206 and from 0.274 to 0.239 for sibling and child-parent respectively, whereas the adequacy of the model was significantly improved. Additional adjustment for rs2000999 for the components of variance attributable to additive genetic effects, shared household effects and residual environmental factors (Table 3, model 3) significantly improved the likelihood function and the proportion of phenotypic variability accounted for by genetic effects decreased (26.0% to 14.2%, in comparison to model 2). Moreover, the component attributable to household factors increased (11.6% to 14.7%). We thus determined that rs2000999 is the major genetic determinant of Hp levels accounting for 11.8% of Hp global variance and 45.4% of the genetic variance of this trait.

In order to assess the degree of independence of rs2000999 from the *HP* rs72294371 ‘common polymorphism’ (Data S1), we genotyped the latter in the GWAS first stage children (SFS cohort) by using a PCR-based method and a gel reading. Only a subset of 341 out of 631 samples was successfully genotyped after independent readings by two readers. In this sample set, we found no evidence for LD between *HP* ‘common polymorphism’ and rs2000999 (r^2^ = 0.135) nor with the other SNPs that were genotyped by the Illumina array within the 218 kb LD block that includes both *HP* ‘common polymorphism’ and rs2000999 (0.001<r^2^<0.137; *N* = 31 SNPs). Furthermore, the *HP* ‘common polymorphism’ (minor allele frequency [MAF] = 0.46) and rs2000999 (MAF = 0.20) were both highly associated with Hp levels, as expected (*P* = 4×10^−7^ and *P* = 1×10^−7^, respectively). When both variants were included in the same regression model, we found that they significantly and independently contributed to the increased Hp levels (*P_HP rs72294371 ‘common polymorphism’_* = 0.001 and *P_rs2000999_* = 5×10^−5^) indicating that the association with rs2000999 would be novel and not redundant with the *HP* ‘common polymorphism’. However, it is noteworthy that despite strong efforts, we did not succeed by far in genotyping all samples. We used two other technologies: a pre-designed TaqMan copy number assay (Applied Biosystems) and a PCR-based method with another design than previously used. Unfortunately, we did not find a good concordance (<70%) between the three methods. We conclude that given the state of art, we cannot definitively conclude that the present signal of association is not related to the *HP* ‘common polymorphism’ genotype.

In order to validate our main results, we secondary assessed the effect of SNP rs2000999 on *HP* gene expression in subcutaneous adipose tissue sample from 194 non-obese subjects ascertained from the Swedish SibPair cohort (Data S2). We found a significant contribution of rs2000999 to *HP* expression (β = 0.23±0.08; *P* = 0.007; *P_Bayesian_* = 0.006).

We finally assessed by additive model the effect of SNP rs2000999 on total, HDL and low-density lipoprotein (LDL) cholesterol, Apolipoproteins A1 and B in five independent European pediatric cohorts totaling 8,789 children. Total cholesterol was ln-transformed and we normalized the LDL cholesterol by computing the square root. All measurements were adjusted for gender, age (excepting the NFBC1986) and z-score BMI. Our data showed that rs2000999, with A as allele effect, was associated with total cholesterol (β = −0.011, SE = 0.003, *P* = 0.002; Table 5) and LDL-cholesterol (β = −0.017, SE = 0.004, *P* = 0.0008; Table 5). The association with HDL-cholesterol and Apolipoproteins A1 and B are displayed in Table S2.

**Table 5:** Association of rs2000999 with lipid traits.

				Total cholesterol			LDL-cholesterol	
	N	MAF	β	Standard error	p-value	β	Standard error	p-value
SFS children	1,644	0.202	−0.003	0.008	0.73	0.0004	0.010	0.967
Obese Children	1,015	0.216	−0.008	0.011	0.439	−0.009	0.013	0.507
GENDAI	419	0.253	−0.023	0.012	0.055	−0.031	0.013	0.022
NFBC1986	5,310	0.186	−0.013	0.004	0.002	−0.022	0.006	8.96E-05
Verona Cohort	401	0.200	−0.011	0.014	0.446	−0.011	0.016	0.515
Overall meta-analysis	8,789		−0.011	0.003	0.002	−0.017	0.004	0.0008

N: sample size; MAF: Minor Allele Frequency; β:beta coefficient for the effect allele A.

## Discussion

We first determined in 656 nuclear families that 26% of the Hp plasma level variance was under genetic control. Then, using a GWAS in 631 children from the same population and replicating in three independent populations, we identified rs2000999 as the major genetic determinant of Hp levels. This genetic variant alone explained 45.4% of the genetic variance of this trait (11.8% of Hp global variance). SNP rs2000999 is located in the intronic region of *HP* gene, in a region previously believed to be the *HPR* gene (encoding the haptoglobin-related protein) which shares more than 90% nucleotide sequence homology with *HP* [30]. It is 17 kb apart a duplication of 59 α chain amino acid residues resulting to an intragenic duplication of 1.7 kb and which is known as the *HP* ‘common polymorphism’ [31].

Our study shows that SNP rs2000999 also modulated expression levels of the Hp mRNA in human adipose tissue suggesting that this SNP (or a SNP in very strong LD with this one) is indeed functional. It is noteworthy that SNP rs2000999 has been previously reported to associate with total cholesterol in 4,200 adults from the EUROSPAN consortium [32] and with both total and LDL-cholesterol in 100,000 adults of European and non-European ancestry [33]. Interestingly, we confirmed the effect of this SNP on these lipid traits in European children.

Increased plasma levels of several inflammatory markers correlate with higher incidence and prognosis of various cardiovascular diseases [34], [35], [36], [37]. Hp level measurement has been recently shown to improve the predictive information for major cardiovascular events [29]. As rs2000999 is also associated with lipid levels, this marker links inflammation and cardiovascular risk. It is noteworthy that the impact of rs2000999 association on lipids occurs early in life and is consistent with previous findings that the precursors of cardiovascular diseases originate in childhood [38], [39].

Interestingly, the effect of rs2000999 on Hp levels is more important in our discovery cohort which includes healthy children having low Hp concentration (0.65 g/L±0.39). As shown in the analyses for other diseases [40], the statistical power of GWAS can be increased in healthy homogeneous controls.

In addition, the effect of aging and of the environment is minimized in children. Then, by using healthy pediatric populations, we were able to assess more accurately the effect of the SNP rs2000999 on Hp levels.

We tried to assess the degree of independence between rs2000999 and the *HP* ‘common polymorphism’. Three different methods were evaluated to genotype the *HP* ‘common polymorphism’ in our whole GWAS sample set. Unfortunately, we found no concordance between the three methods, which underlie a major difficulty to carry out an accurate genotyping of this polymorphism. Even if this difficulty was not clearly discussed and not published to our knowledge, it is admitted in the scientific field and it should also be present in the clinical diagnosis setting. In contrast, SNP rs2000999 can be accurately and easily genotyped.

Our findings should be further replicated in non-European adults, especially in those affected by infectious diseases. More generally, rs2000999 should be assessed in cohorts of patients affected by the large variety diseases associated with Hp levels. It is not a trivial task, as Hp is a trait that has been infrequently measured in cohorts used for genetic studies. Given the major effect of rs2000999 on Hp gene expression and on Hp levels, Mendelian randomization approach would be of interest to test the causative effect of this SNP on infectious and non-communicable phenotypes in order to assess its clinical relevance.

## Materials and Methods

### Ethics Statement

All the populations involved in the present study were recruited in accordance with the latest version of the Declaration of Helsinki for Ethical Principles for Medical Research Involving Human Subjects. All participants and their parents gave a written informed consent. Genetic studies protocols were approved by the local ethics committees for the protection of subjects for biomedical research: the Comité Consultatif de Protection des Personnes dans la Recherche Biomédicale (CCPPRB).

### Study populations

#### The STANISLAS Family Study (SFS)

The SFS is a 10-year longitudinal survey involving 1,006 volunteer families of European ancestry whose members were free of chronic disease (cardiovascular or cancer) with recruitment taking place from 1993–95 [41]. The SFS samples and data are part of the Biological Resources Centre (BRC) “Interactions Gène-Environnement en Physiopathologie CardioVasculaire” (IGE-PCV) in Nancy, France. Genome-wide genotyping was performed on a subset of 631 unrelated children (mean age 11.93 years [11.76–12.11]) constituting the discovery cohort [42] after screening for latent population substructure (Data S2). The 2,957 remaining individuals after quality control were analysed in the replication studies (mean age 29.84 [29.38–30.30]). Hp levels, BMI and the cardiovascular risk traits including total, high density lipoprotein (HDL) and low density lipoprotein (LDL)-cholesterol (calculated by the Friedewald formula [43]), Apolipoprotein A1 and B were available for all participants.

#### Obese Children

We studied obese children (defined as BMI>97^th^ percentile for age and sex according to a French cohort [44]) ascertained from 449 nuclear families with at least one obese offspring, recruited in the Paediatric Endocrine Unit of Jeanne de Flandres Hospital of Lille, France or through a national media campaign. We analyzed 1,015 children (mean age 11.07 years [10.86–11.27]) for whom Hp, BMI, total, HDL and LDL-cholesterol, Apolipoprotein A1 and B measurements were available.

#### The GeNe and Diet Attica Investigation (GENDAI)

The GENDAI pediatric cohort was recruited from children living in the Attica region of Greece [45]. From November 2005 to June 2006, 1,138 peri-adolescent children were recruited from randomly selected elementary schools of Attica. We analyzed 419 children (mean age 11.16 years [11.10–11.23]) for whom Hp, BMI, total, HDL and LDL-cholesterol, Apolipoprotein A1 and B measurements were available.

#### The Northern Finland 1986 Birth Cohort (NFBC1986)

The NFBC1986 is a prospective birth cohort including all Finnish mothers of European ancestry with children whose expected date of birth fell between July 1, 1985 and June 30, 1986 in the two northernmost provinces in Finland [46]. Clinical examination at 15–16 years follow-up was conducted between August 2001 and June 2002. All cohort members living in Finland with known address (n = 9,215) were invited, and 6,798 participated (74%). We analyzed 5,310 adolescents successfully genotyped in the NFBC1986 cohort for whom BMI, total, HDL and LDL-cholesterol, Apolipoprotein A1 and B measurements were available.

#### The Verona cohort

The Verona cohort consists of Italian children recruited from the general population of Verona, Italy, whose families were randomly chosen from the registry office database of the town, and contacted by post. We analysed 401 children (mean age 10.90 years [10.75–11.04]) successfully genotyped for whom at least BMI, total, HDL and LDL-cholesterol, Apolipoprotein A1 and B measurements were available.

#### The SibPair cohort

The SibPair cohort comprises 154 nuclear families (732 subjects) from Sweden, each containing an obesity-discordant sib pair (at least 10 kg/m^2^ difference in BMI). Gene expression and genetic variation were analysed in 194 non-obese subjects from the SibPair cohort.

### Genotyping

Genomewide genotypes were generated for the 631 unrelated SFS children using the Illumina Human CNV370-Duo array [42]. Briefly,750 ng of genomic DNA was processed using Illumina's protocol for the BeadStation genotyping platform (Illumina), followed by GenCall software analysis(Illumina) to automatically cluster, call genotypes, and assign confidence scores using the GenTrain clustering algorithm (Illumina). We discarded a total of 2,552 SNPs due to the following reasons: extreme Hardy-Weinberg disequilibrium (*P*<0.001), low genotyping call rates (<95%) or low minor-allele frequencies (<1%). We retained 318,237 SNPs for analysis. Genomic control λ_GC_ was 1.01.

We used the Applied Biosystems SNPlex™ technology to replicate the association of genome-wide significant genetic variants in the SFS replication set, obese children and GENDAI, NFBC1986 and Verona cohorts.

SNPlex is based on the Oligonucleotide Ligation Assay (OLA) combined with multiplex PCR target amplification and was carried out as per the manufacturer's instructions (http://www.appliedbiosystems.com). Allelic discrimination was performed by capillary electrophoresis analysis using an Applied Biosystems 3730xl DNA Analyzer and GeneMapper 3.7 software. Genotyping call rate was above 95% in all populations studied and genetic variants were in HW equilibrium (p>0.001).

We used a PCR-based method [47] to genotype for the *HP* ‘common polymorphism’ in the 631 children of the discovery cohort (SFS cohort) in order to determine any linkage disequilibrium with regard to genome-wide significant variants identified in the analysis. Only genotypes that were concordant following a double blind genotyping call by two independent readers were retained for statistical analyses (*N* = 341). Two additional genotyping methods for *HP* ‘common polymorphism’ were used in order to validate the above-method: a custom TaqMan copy number assay (Applied Biosystems) following the manufacturer's recommendations and another PCR-based method using the following oligonucleotide primers : 5′-CTCTCCTTTCTCCCTTCCTGTC-3′ and 5′-TTTATCCACTGCTTCTCATTGT-3′. We didn't obtain correspondence between the banding patterns and the Hp genotypes.

### Haptoglobin measurement

Blood samples were collected between 8:00 and 9:00 am or 11:30 and 12:30 pm by venipeuncture after overnight fasting. Hp protein levels were measured in blood plasma samples by high sensitivity immunophotometry analyses using the BN™II Siemens analyzer (Siemens, Marburg, Germany) and Siemens reagents and following the manufacturer's instructions.

### Lipids measurements

Total cholesterol, HDL-cholesterol and apolipoproteins A1 and B were assayed using enzymatic methods (AU640 [Olympus, Watford, UK]) and LDL-cholesterol was calculated using the Friedewald formula [43].

### Statistical analyses

#### Heritability estimate of Hp levels in the SFS

Intra-familial correlations were estimated by using maximum likelihood techniques [48] with and without adjustment for covariates. This statistical approach allowed adjustment for covariates within models, simultaneously and separately for fathers, mothers, sons and daughters. The significance of various familial correlations, or sex and generation differences in correlations, was tested using the log-likelihood ratio test. Correlations were computed under two sets of hypotheses: gender effects on correlations for parents and children and no gender effect for all correlations.

Variance component analysis was applied in order to assess the relative contributions of genetic, common household factors and individual specific environment in familial aggregation of serum haptoglobin concentrations. The variable used to estimate variance component was adjusted for age and BMI, separately for fathers, mothers, sons and daughters. The analysis was conducted by using a multivariate normal model for pedigree analysis as described by Lange and colleagues [49], [50]. with the software FISHER, which also performed tests of goodness-of-fit of the underlying multinormal distribution. The general model assumed that the studied trait was the result of the sum of three independent random components: a polygenic component (G) representing additive genetic factors, household factors common to individuals within a family (H) and unmeasured environmental factors particular to an individual (including measurement error) (E). These three components were assumed to be normally distributed with mean equal to 0 and variance equal to σ^2^G, σ^2^H and σ^2^E, respectively.

The hypothesis of no polygenic component or no household effect was checked by comparing a model including σ^2^G, σ^2^H and σ^2^E with a model including only σ^2^H and σ^2^E or σ^2^G and σ^2^E, respectively. In addition, possible effects of covariates (age and BMI) and genome-wide significant variants' allelic frequency on these variance components were tested.

Comparison of nested models was based on the likelihood ratio criteria. Eventually, the best parsimonious model was selected. The percentage contributions of the three components, additive genetic factors (heritability), household factors and residual environmental, to residual phenotypic variance (after adjustment for covariates) were determined.

#### Genome-wide association and replication analyses

We carried out genome-wide association and replication analyses on Hp levels using linear mixed regression models under the additive genetic model with one degree of freedom, adjusting for age, gender and BMI and using PLINK [51]. The summary statistics were combined in the meta-analyses (Data S2), using the inverse normal method with equal weight for each population. In this method, P values of each study are transformed into their inverse normal z score and the weighted sum, over all studies, is compared to a normal N (0, 1), provided the sum of squared weights equals 1. The estimates of variants effects on Hp and their standard errors for each separate analysis were combined in the meta-analysis using the weighted inverse normal method, and the overall effect and its confidence interval were estimated using the inverse variance method implemented in the ‘meta.summaries’ function of the R RMETA package (http://cran.r-project.org/web/packages/rmeta/index.html). No major heterogeneity in effects was observed (P<0.02). The same mixed model and the same software were used to analyse the association of genetic variants with lipid traits.

#### Gene-expression investigation

To investigate the effect of genome-wide significant variants on gene expression (Data S2), we used data from 194 non-obese individuals from the SibPair cohort [52]. Gene expression data for *HP* was measured in subcutaneous adipose tissue [53] from 347 siblings using the Affymetrix Human U133 Plus 2.0 platform (208470_s_at and 208471_at, respectively). DNA was isolated from peripheral blood and genotypes were generated using Illumina 610-Quad arrays.

We used a linear mixed model (Pinheiro and Bates, 2000) to assess association of SNPs with gene expression. Log-transformed expression level was regressed on the random-effect term, which accommodates the family pedigree structure, and on the fixed-effect terms i.e. sex, age, BMI level and the SNP of interest (recoded as 0 = AA; 1 = AG; 2 = GG according to an additive model). Analysis was carried out using the R function lmer() (package *lme4*) with p-values obtained from the *t*-statistic.

Significance of the fixed effects was further investigated in the Bayesian set-up using the R function mcmcsamp() (package *lme4*) that generates Monte Carlo Markov Chain samples from the posterior distribution of the parameters of a linear mixed model. The prior on the fixed effects parameters is taken to be locally uniform while the prior on the variance-covariance matrices of the random effects is taken to be the locally non-informative prior. Based on 100,000 samples drawn from the posterior distribution, we calculated the smallest *p* such that the (1−*p*) credible interval does not contain the value 0. This parameter was finally used to assess the p-value obtained from the *t*-statistic: if smaller than *p*, it was considered anticonservative and its value discarded.

## Supporting Information

Data S1 ***HP* rs72294371 ‘common polymorphism’ flanking sequence.** Source: 1000Genome. (http://browser.1000genomes.org/Homo_sapiens/Variation/Summary?r=16:72090747-72091766v=rs72294371vdb=variationvf=13544249).
(DOC)

Data S2 **Supplementary Methods. 1. Screening of latent population substructure. 2. Conditional analysis. 3. Meta-analysis. 4. Gene-expression analysis.**
(DOC)

Table S1 **Conditional regression within the recombination hotspot around rs2000999.** UNADJ: unadjusted p-values, SNP: single nucleotide polymorphism, add: additive model.
(XLS)

Table S2 **Association of rs2000999 with HDL-cholesterol and Apolipoproteins A1 and B.** N: sample size; MAF: Minor Allele Frequency; β: beta coefficient for the effect allele A.
(DOC)
