# Supplementary material for: A Genome-Wide Association Study Identifies rs2000999 as a Strong Genetic Determinant of Circulating Haptoglobin Levels
Source: PLoS One. 2012 Mar 5;7(3):e32327. doi: 10.1371/journal.pone.0032327 (PMC3293812; doi:10.1371/journal.pone.0032327)
Supplement: Data S1 — HP rs72294371 ‘common polymorphism’ flanking sequence. Source: 1000Genome. (http://browser.1000genomes.org/Homo_sapiens/Variation/Summary?r=16:72090747-72091766v=rs72294371vdb=variationvf=13544249). (DOC) [file pone.0032327.s001.doc]

**Supplementary Data S1: *HP* rs72294371 ‘common polymorphism’ flanking sequence**

Reference AGTGAGTCTTGCTGTCCTGGCACTGCTCTAAGGGCTTTATACTTATTTGCTCACTTAGTC

dbSNP AGTGAGTCTTGCTGTCCTGGCACTGCTCTAAGGGCTTTATACTTATTTGCTCACTTAGTC

Reference CTCACAGTATCCCTCTGAACAGAGTTTATTGTTTTCACTTTGCTGATAAGGAAACTGAGG

dbSNP CTCACAGTATCCCTCTGAACAGAGTTTATTGTTTTCACTTTGCTGATAAGGAAACTGAGG

Reference CACAGACAGGTTGAGTATCTTGCCCAAATTCAGGCAGCCTGTAAGAGGCAGAGTCAGGAT

dbSNP CACAGACAGGTTGAGTATCTTGCCCAAATTCAGGCAGCCTGTAAGAGGCAGAGTCAGGAT

Reference TTGAACCCTGAGCCCTCCCTGTACTGCTTGGCTGTGACCGCCATGACCACAGTGTGTTCT

dbSNP TTGAACCCTGAGCCCTCCCTGTACTGCTTGGCTGTGACCGCCATGACCACAGTGTGTTCT

Reference GCTGGGCTTAACTGGTGTCCAGGCACTTGGCTTCCAGCACAGCACTCTTTCCCTTCCTCC

dbSNP GCTGGGCTTAACTGGTGTCCAGGCACTTGGCTTCCAGCACAGCACTCTTTCCCTTCCTCC

Reference TTCTCATATTCTCTCTCCTTTCTCCCTTCCTGTCTGCCTCCTTTCTTCTTCTTCTTTTTA

dbSNP TTCTCATATTCTCTCTCCTTTCTCCCTTCCTGTCTGCCTCCTTTCTTCTTCTTCTTTTTA

Reference ATTCTTCTCCTTAAATGCCTTCTCACTCTGCTCTGGGTGC[(LARGEDELETION)]---

dbSNP ATTCTTCTCCTTAAATGCCTTCTCACTCTGCTCTGGGTGC[(LARGEDELETION)]AGA

Reference CTC-A-TT-TC-TT-G-C-C--TT-TTG--TTT-----CAGGAGTATACACCTTAAATGA

dbSNP CTTGACTTCTCCTTTGGCTCACTTCTTGCCTTTTGTTTCAGGAGTGTACACCTTAAACAA

Reference TAAGAAGCAGTGGATAAATAAGGCTGTTGGAGATAAACTTCCTGAATGTGAAGCAGGTGG

dbSNP TGAGAAGCAGTGGATAAATAAGGCTGTTGGAGATAAACTTCCTGAATGTGAAGCAGGTGG

Reference GTGCTGAGCACTGAGCACTTAAGAGAGCAGGCAGGCGTCCAGCGGGGAACGTCCTAGAGG

dbSNP GTGCTGAGCACT------T-AAGAGAGCAGGCAGGCGTCCAGCGGGGAACGTCCTAGAGG

Reference CACAGCCTTCCAGTGCGGCTTCCTCTGAGCACACAAGAGCCAGGAGGAGGGATGTGGGAG

dbSNP CACAGCCTTCCAGTGCGGCTTCCTCTGAGCACACAAGAGCCAGGAGGAGGGATGTGGGAG

Reference AACCGCAGCTGGCCAGGGAGAGACTTAAGCAGTTAGGTGATGACTCCCTAAGGGTCACCA

dbSNP AACCGCAGCTGGCCAGGGAGAGACTTAAGCAGTTAGGTGATGACTCCCTAAGGGTCACCA

Reference AGGGTCTTGTTCATTGGGGCCTGAAGGGCACTGGCTGAATCCACTGTCGGCA-CTGCCCA

dbSNP AGGGTCTTGTTCATTAGGGCCTGAAGGGCACTGGCTGAATCCATTGTCTACATC-GCCCA

Reference CAGATCAGGAGAGCCTGTGCATACAGAGAGCCTGCTAGAAAGCCCTGGGTCTAAGGA

dbSNP CAGATTAGGAGAGCCTGTGCATACAGAGAGCCTGCTAGAGAGCC-------------
