# Supplementary material for: A Genome-Wide Association Study Identifies rs2000999 as a Strong Genetic Determinant of Circulating Haptoglobin Levels
Source: PLoS One. 2012 Mar 5;7(3):e32327. doi: 10.1371/journal.pone.0032327 (PMC3293812; doi:10.1371/journal.pone.0032327)
Supplement: Data S2 — Supplementary Methods. 1. Screening of latent population substructure. 2. Conditional analysis. 3. Meta-analysis. 4. Gene-expression analysis. (DOC) [file pone.0032327.s002.doc]

**Supplementary Data S2: Supplementary Methods**

1. **Screening of latent population substructure.**

The pediatric sample used for stage 1 genome-wide association study and extracted from the STANISLAS Family Study was screened for latent population substructure using STRUCTURE [1]. Sixty-two population outliers with less than 90% European ancestry were detected and subsequently excluded from the analysis. To correct for variance inflation owing to systematic genotyping errors or subtle subpopulation structure during genome-wide association studies, P-values were adjusted using a correction for the inflation of chi-square distribution (median of the observed distribution/0.675²) [2]. False Discovery Rates were calculated with R’s p.adjust procedure using the method of Benjamini and Hochberg [3] as previously described [4].

1. **Conditional analysis**

We selected SNPs with independent effects by running a conditional regression for the top 6 genetic variants in a 318k LD-block associated with Hp levels: rs2335712; rs1092825; rs4788597; rs3764310; rs2000999 and rs8060878; to which we added the SNPs within the recombination hotspot around our higher hit: rs2000999.


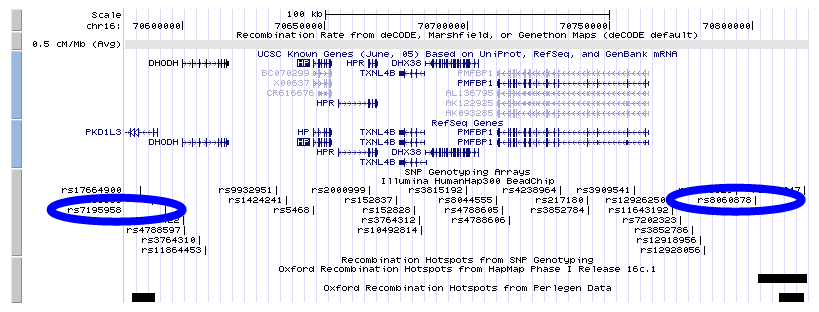


The corresponding SNPs are listed below:

| Chromosome | SNP | A1 | A2 | Minor Allele Frequency |
| --- | --- | --- | --- | --- |
| 16 | rs7195958 | A | G | 0.491918 |
| 16 | rs3213422 | A | C | 0.491894 |
| 16 | rs4788597 | T | C | 0.33914 |
| 16 | rs3764310 | C | T | 0.302351 |
| 16 | rs11864453 | T | C | 0.387085 |
| 16 | rs9932951 | A | G | 0.428781 |
| 16 | rs1424241 | T | C | 0.194883 |
| 16 | rs5468 | G | T | 0.0991888 |
| 16 | rs2000999 | A | G | 0.208644 |
| 16 | rs152837 | G | A | 0.0749631 |
| 16 | rs152828 | A | G | 0.117561 |
| 16 | rs3764312 | C | A | 0.384091 |
| 16 | rs10492814 | A | G | 0.40986 |
| 16 | rs3815192 | C | A | 0.0511322 |
| 16 | rs8044555 | C | T | 0.241354 |
| 16 | rs4788605 | T | G | 0.170839 |
| 16 | rs4788606 | G | T | 0.151103 |
| 16 | rs4238964 | C | A | 0.41839 |
| 16 | rs217180 | A | G | 0.0674487 |
| 16 | rs3852784 | G | A | 0.439249 |
| 16 | rs3909541 | T | C | 0.448557 |
| 16 | rs12926250 | T | G | 0.0962963 |
| 16 | rs11643192 | A | C | 0.403155 |
| 16 | rs7202323 | G | T | 0.229948 |
| 16 | rs3852786 | G | A | 0.496684 |
| 16 | rs12918956 | C | T | 0.455979 |
| 16 | rs12928056 | A | C | 0.0997067 |
| 16 | rs2023929 | T | C | 0.138251 |
| 16 | rs8060878 | G | A | 0.466543 |
| 16 | rs811047 | C | T | 0.400891 |

The corresponding results are displayed in Supplementary Table S1.

1. **Meta-analysis**

For the meta-analysis of results from the SFS, Obese children and GENDAI, we used a fixed effects inverse-variance weighted meta-analysis technique. Beta estimates were weighted by their inverse variance and a combined estimate was obtained by summing the weighted betas and dividing by the summed weights. Strand alignment was verified across all studies prior to meta-analysis.

1. **Gene-expression analysis**

To investigate the effect of rs2000999 on gene expression we used data from 149 obesity-discordant families included in the SibPair cohort [5]. Gene expression data for HP and HPR were measured in subcutaneous adipose tissue from 347 siblings using the Affymetrix Human U133+2.0 platform (208470_s_at and 208471_at, respectively). Genotypes for rs2000999 were obtained through Illumina 610-Quad arrays in peripheral blood.

We used a linear mixed model [6] to assess association of rs2000999 with HP and HPR gene expressions. Log-transformed gene expression was regressed on the random-effect term, that accommodates the family pedigree structure, and on the fixed-effect terms i.e sex, age, BMI level and the rs2000999 genotype (recoded as 0=AA; 1=AG; 2=GG according to an additive model). Analysis was carried out using the R function lmer (package lme4) with p-values obtained from the t-statistic.

Significance of the fixed effects was further investigated in the Bayesian set-up using the R function mcmcsamp (package lme4) that generates Monte Carlo Markov Chain samples from the posterior distribution of the parameters of a linear mixed model. The prior on the fixed effects parameters is taken to be locally uniform while the prior on the variance-covariance matrices of the random effects is taken to be the locally non-informative prior. Based on 100,000 samples drawn from the posterior distribution, we calculated the smallest p such that the (1 - p) credible interval does not contain the value 0. This parameter was finally used to assess the p-value obtained from the t-statistic: if smaller than p, it was considered anticonservative and its value disbelieved.

HPR gene expression was not associated with rs2000999 in the SibPair cohort, while association was observed for HP (p-value = 0.03).

Obesity-induced inflammatory changes in adipose tissue lead to strong increase in HP levels in our sample, determining significant differential expression of HP between non-obese and obese subjects (p-value from Wilcoxon rank sum test = 9.3 x 10^-15^).

**
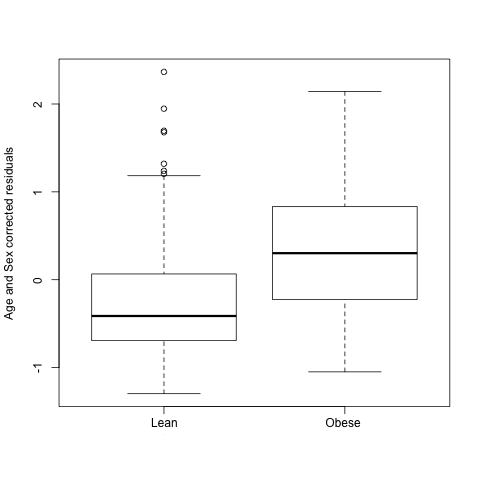
**

**Box-plot of the age and sex adjusted residuals for HP transcript 208470_s_at in the lean and obese subjects.**

The association observed between HP levels and BMI in the linear mixed model was mainly determined by their correlation within the subgroup of obese subjects (p-values for BMI = 0.09 and 1.4 x 10^-09^ using separate linear mixed model for lean and obese subjects, respectively). Taking into consideration the heterogeneous effect of BMI on HP gene expression in this sample, we carried out the association analysis with rs2000999 using the subgroup of 194 non-obese subjects detecting significant additive effect of the G allele of 0.23 with SE 0.08 (p-value = 0.007 and Bayesian p = 0.0064).

**References**

1. Pritchard JK, Stephens M, Donnelly P. Inference of population structure using multilocus genotype data. Genetics. 2000 Jun;155(2):945-59.
2. Devlin B, Roeder K. Genomic control for association studies. **Biometrics**. 1999 Dec;55(4):997-1004.
3. Benjamini Y, Hochberg Y. Controlling the False Discovery Rate: A Practical and Powerful Approach to Multiple Testing. J R Stat Soc Ser B Stat Methodol 57, 289-300 (1995).
4. Meyre D, Delplanque J, Chèvre JC, Lecoeur C, Lobbens S, Gallina S, Durand E, Vatin V, Degraeve F, Proença C, Gaget S, Körner A, Kovacs P, Kiess W, Tichet J, Marre M, Hartikainen AL, Horber F, Potoczna N, Hercberg S, Levy-Marchal C, Pattou F, Heude B, Tauber M, McCarthy MI, Blakemore AI, Montpetit A, Polychronakos C, Weill J, Coin LJ, Asher J, Elliott P, Järvelin MR, Visvikis-Siest S, Balkau B, Sladek R, Balding D, Walley A, Dina C, Froguel P. Genome-wide association study for early-onset and morbid adult obesity identifies three new risk loci in European populations. Nat Genet. 2009 Feb;41(2):157-9.
5. Walley AJ, Jacobson P, Falchi M et al. Differential co-expression analysis of obesity-associated networks in human subcutaneous adipose tissue. Int J Obes (in press).
6. Pinheiro JC, Bates DM. Mixed-Effects Models in S and S-PLUS. New York: Springer. (2000)
