# Supplementary material for: A Genome-Wide Association Study Identifies rs2000999 as a Strong Genetic Determinant of Circulating Haptoglobin Levels
Source: PLoS One. 2012 Mar 5;7(3):e32327. doi: 10.1371/journal.pone.0032327 (PMC3293812; doi:10.1371/journal.pone.0032327)
Supplement: Table S2 — Association of rs2000999 with HDL-cholesterol and Apolipoproteins A1 and B. N: sample size; MAF: Minor Allele Frequency; β: beta coefficient for the effect allele A. (DOC) [file pone.0032327.s004.doc]

**Supplementary Table S2: *Association of rs2000999 with HDL-cholesterol and Apolipoproteins A1 and B***

|  |  |  | **HDL-cholesterol** | | | **ApoA1** | | | **ApoB** | | |
| --- | --- | --- | --- | --- | --- | --- | --- | --- | --- | --- | --- |
|  | N | MAF | β | Standard error | p-value | β | Standard error | p-value | β | Standard error | p-value |
| SFS children | 1.644 | 0.202 | -0.0013 | 0.0112 | 0.95 | -0.0422 | 0.0193 | 0.85 | 0.001 | 0.016 | 0.25 |
| Obese Children | 1.015 | 0.216 | 0.005 | 0.0131 | 0.23 | 0.009 | 0.0256 | 0.65 | -0.0208 | 0.0227 | 0.84 |
| GENDAI | 419 | 0.253 | 0.0011 | 0.0149 | 0.54 | 0.0062 | 0.0235 | 0.48 | -0.0089 | 0.0231 | 0.98 |
| NFBC1986 | 5.31 | 0.186 | 0.0031 | 0.0048 | 0.09 | -0.0075 | 0.0104 | 0.12 | 0.0185 | 0.009 | 0.26 |
| Verona Cohort | 401 | 0.200 | 0.0159 | 0.0148 | 0.54 | -0.0536 | 0.0344 | 0.23 | -0.0007 | 0.0301 | 0.84 |
